# Pilot study on the use of a multimorbidity index in patients receiving home parenteral nutrition

**DOI:** 10.1002/ncp.70034

**Published:** 2025-09-16

**Authors:** Michael M. Rothkopf, Mohan Pant, Zachary Rothkopf, Rebecca Brown, Jamie Haselhorst, Debbie L. Stevenson, Andrew DePalma, Michael Saracco

**Affiliations:** ^1^ Rutgers/New Jersey Medical School Newark New Jersey USA; ^2^ Epidemiology, Biostatistics & Environmental Health Old Dominion University Norfolk Virginia USA; ^3^ St. Luke's University Hospital Bethlehem Pennsylvania USA; ^4^ Sea Meadows, LLC, Brick New Jersey USA

**Keywords:** CIRS, home TPN, HPN, Length of Stay, LOS, multimorbidity, parenteral nutriton

## Abstract

**Background:**

Home parenteral nutrition (HPN) patients often have multiple comorbidities. A validated multimorbidity index (MMI) could help determine resource needs and risks with HPN. We evaluated MMI scoring to determine if it predicted HPN resource use and outcomes.

**Methods:**

We performed a secondary analysis of 60 HPN patients from a previous study based on Cumulative Illness Rating Scale (CIRS) scoring. We examined three variables: PN formula changes, hospitalizations, and hospital length of stay (LOS). Density plots were produced to select a CIRS score cutoff value. Spearman correlations among the three variables of interest were computed. The data were then subjected to Wilcoxon rank sum tests and negative binomial regression models to determine if the measured variables differed significantly between the groups.

**Results:**

In 60 HPN patients, CIRS scores ranged from 9 to 25 with a mean ± SD of 17.0 ± 3.85. Patients with CIRS scores ≥ 17 had higher rates of the three variables than those with CIRS scores < 17 (PN formula changes = 367 vs 297, hospitalizations = 19 vs 12, and total hospital LOS days = 122 vs 100). Although these raw data did not differ significantly, negative binomial regression analysis indicated that the interaction of total hospital LOS and PN formula changes was significantly higher in patients with a CIRS score ≥ 17 than a CIRS score < 17.

**Conclusion:**

CIRS data from HPN patients showed a trend wherein higher multimorbidity scores were associated with PN changes and LOS. This approach requires further study and validation.

## Graphical abstract

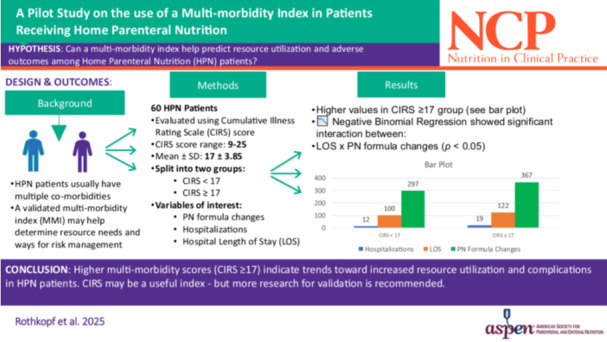

## INTRODUCTION

Home parenteral nutrition (HPN) patients often have concomitant medical conditions that impact their care and confound outcomes. The existence of multimorbidity in HPN patients may increase their risk for serious complications (ie, catheter‐related bloodstream infections, venous thrombosis, metabolic imbalances, bone disease, kidney stones, and liver disease).^1^, ^2^ However, research has yet to thoroughly address the role of multimorbidity in relation to HPN risk.

The risk of HPN complications varies widely. Previous studies have suggested a relationship among patient demographics, length of functional bowel, duration of HPN therapy, and macronutrient content.^3^, ^4^ HPN risks can be reduced by careful monitoring and patient education.^5^

An accurate, validated method for determining multimorbidity in HPN patients would be beneficial for practitioners, researchers, and payors. A multimorbidity index (MMI) could be used to better determine the anticipated needs of each HPN patient. It could improve communication between providers, better allocate patient care resources, and more clearly quantify the complexity of care to payors. From a research perspective, a validated HPN MMI could assist in comparisons of patient outcomes, use, and quality of life analysis.

We previously studied the effect of a physician nutrition expert–led multidisciplinary nutrition support team (MNST) in a quality‐improvement project for HPN (QIP‐PN).^6^ In that original study, we showed that intervention from an MNST produced measurable improvements in the care of long‐term HPN patients. MNST input improved patients' self‐ assessed overall health and reduced adverse outcomes, rehospitalization, and hospital length of stay (LOS).

In the current study, we performed a secondary analysis measure of the multimorbidity of HPN patients in our previous report.

## MATERIALS AND METHODS

### Choice of MMI

More than 30 methods have been used to measure multimorbidity with no universally agreed upon MMI tool.^7^ Most MMIs use disease counts.^8^, ^9^, ^10^, ^11^, ^12^, ^13^, ^14^, ^15^, ^16^, ^17^, ^18^, ^19^, ^20^, ^21^ Others use diagnostic groups/clusters or drug counts,^22^, ^23^, ^24^, ^25^, ^26^, ^27^, ^28^, ^29^ and one index used physiologic data from pulmonary function testing, imaging, and laboratory results.^30^

For this study, we eliminated consideration of MMIs based on disease counts, drug counts, and physiologic parameters because their listed comorbidities (eg, myocardial infarction, congestive heart failure, peripheral vascular disease, cerebrovascular disease, dementia, and chronic obstructive pulmonary disease) were less applicable to the HPN population. We also sought a tool that was not geriatric focused, as most HPN patients are aged <65 years.^31^ We reviewed studies that reported using the Charlson comorbidity index (CCI) as an MMI for PN patients^32^, ^33^, ^34^ and used the Stirland guide^35^ to assist in selecting a MMI tool. At the conclusion of this process, we selected the Cumulative Illness Rating Scale (CIRS) as the MMI tool for this study.

The CIRS is a well‐validated and reliable index.^36^ It has been shown to be useful for studies determining risks of mortality, re‐admission, or future morbidity.^36^ Of particular interest, the CIRS can be used to quantify chronic disease burden and has been associated with patient outcomes.^37^, ^38^, ^39^ In this pilot study, we used input from members of a multidisciplinary team to score the CIRS on each patient.

### Research question

Is multimorbidity associated with greater resource use or increased adverse outcomes in HPN care? Does it help predict the need for additional resources for specific patients? Lastly, can MMIs help identify patients at greater risk of HPN complications?

This pilot study was conducted as a secondary analysis of data from a QIP‐PN.^6^

### Study group

To examine the value of MMI in HPN patients, we queried the data of 72 long‐term HPN patients who had undergone CIRS scoring.

#### Inclusion and exclusion criteria

All long‐term adult HPN patients treated at designated home infusion provider branches were offered study participation. The inclusion criteria were not limited to diagnosis, age, or sex. Patients were excluded from the study if they did not sign the study consent form, if their treating physician did not sign the study participation agreement, or if they had not completed >90 days of therapy. Sixty patients met the inclusion and exclusion criteria.

### Study interventions

MNST meetings discussed each patient case in an “open‐book” environment. MNST members (employees or consultants to the HPN provider) had access to the patient electronic medical record (EMR) when cases were discussed. Results of patient PN formula changes, hospitalizations, and total hospital LOS were recorded. The protocol was granted institutional review board (IRB) exemption under National Institutes of Health guidelines (45 CFR 46.104(d)(2)) by the Western IRB on September 4, 2019.

### Multimorbidity assessment

MNST members received training on the CIRS rating scale using the method from de Groot et al.^37^ During each patient's initial case presentation, MNST members analyzed their comorbidities. EMR data included hospital discharge notes, homecare admission notes, follow‐up physician and nursing notes, medication reconciliations, emergency department visits, treatment plans, care summaries, and other available data. At least one MNST member had direct clinical experience with the patient. Each patient was presented to the MNST with a brief history of their case and HPN care at their initial case review. A tally of known conditions in each of 15 body systems was performed. Each system received a weighted score of 0–4, as described previously.^36^, ^37^, ^38^, ^39^, ^40^ A CIRS‐specific spreadsheet was developed, which calculated the total CIRS score for each patient. Performance of the CIRS scoring analysis took approximately 30 min per case. We have provided our CIRS spreadsheet form as Table S1.

### Statistical analysis

We used the mean CIRS score of the study group as a cutoff to divide participants into two groups: one with scores below the cutoff and another with scores equal to or above it.

We used a nonparametric statistical approach for comparison of the two CIRS‐based groups on the variables of interest, which were formula changes, hospitalization, and total LOS. Density plots were obtained to show approximate similarity in the distributions of the variables of interest between the two CIRS‐based groups. A correlation matrix was obtained showing Spearman correlations among the three variables of interest to assess the degree of association. Statistical analysis included computation of descriptive statistics in the form of mean, SD, minimum, median, interquartile range, and maximum to summarize the three variables of interest with count data. Additionally, the data were subjected to Wilcoxon rank sum tests and negative binomial regression models to determine if the groups differed significantly on the variables of interest. A negative binomial regression model is considered appropriate when the data are overdispersed, that is, when the conditional variance of the response variable is greater than the conditional mean.^41^ In this study, the variables of formula changes, hospitalization, and total LOS are all overdispersed. In all tests we used a 5% level of significance. Any test with a *P* value < 0.05 was considered significant.

## RESULTS

Among the 60 patients in the study group, the mean ± SD CIRS score was 17 ± 3.85. Using the cutoff point of 17, roughly half of the patients fell into each group: 32 (53%) in the CIRS ≥ 17 group and the remaining 28 (47%) in the CIRS < 17 group.

During the study period, the number of formula changes was higher for patients in the group of CIRS ≥ 17 (*n* = 367) compared with those in the group of CIRS < 17 (*n* = 297). The number of hospitalizations were higher for patients in the group of CIRS ≥ 17 (*n* = 19) compared with those in the group of CIRS < 17 (*n* = 12). Total LOS was numerically higher for patients in the group of CIRS ≥ 17 (*n* = 122 days; 3.81 days/patient) compared with those in the group of CIRS < 17 (*n* = 100 days; 3.57 days/patient) (Table 1). These differences were not statistically significant.

**Table 1:** Homecare use, hospitalization, and hospital length of stay during a 90‐day study period.

		CIRS ≥ 17, *n*		CIRS < 17, *n*
Patients		32		28
PN formula changes		367		297
Total hospitalizations		19		12
Total hospital length of stay		122		100
Hospital days/patient		3.81		3.57

Abbreviations: CIRS, Cumulative Illness Rating Scale; PN, parenteral nutrition.

**Figure 1:**
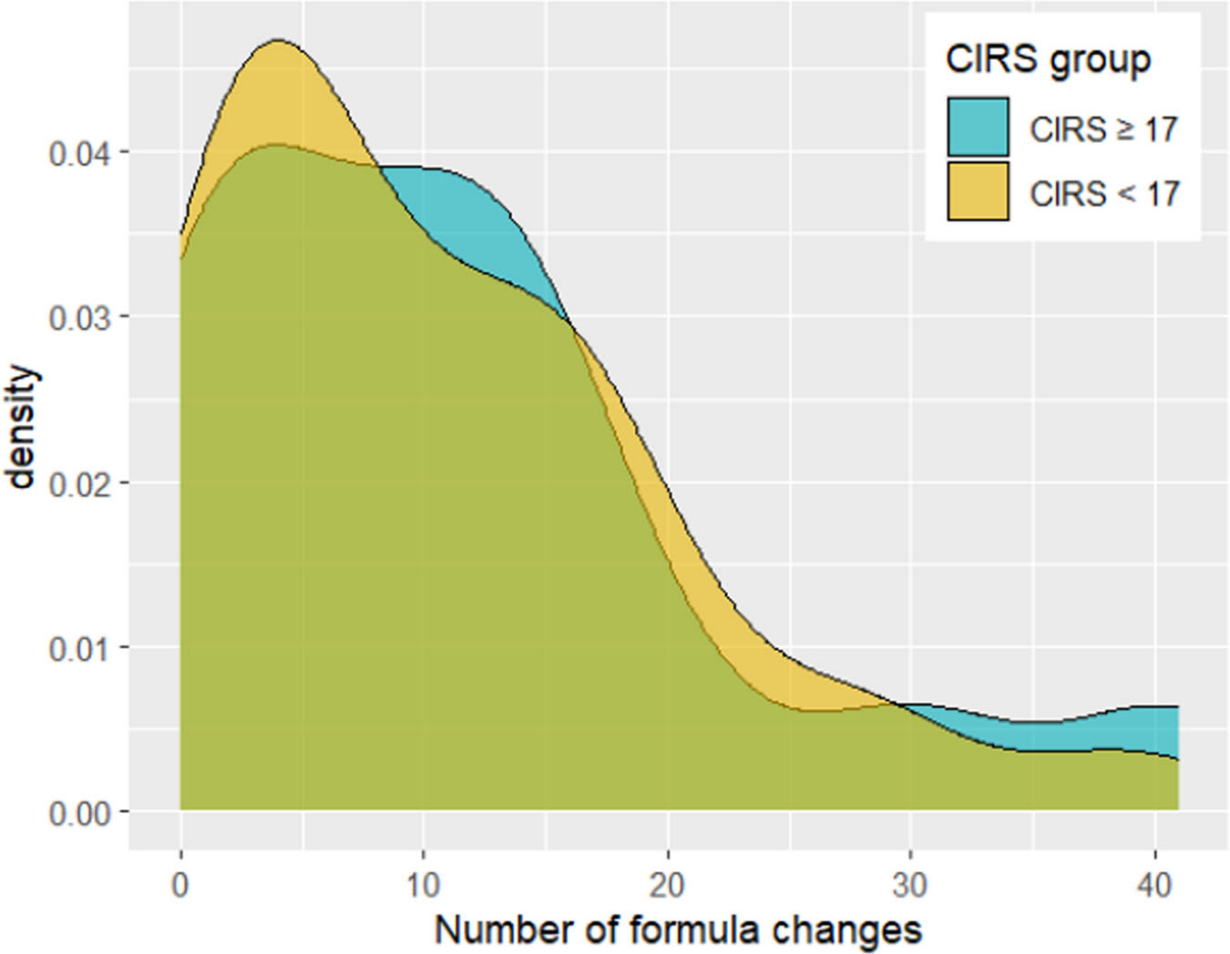
Density plot of the Number of formula changes for the two groups (CIRS ≥ 17 and CIRS < 17). CIRS, Cumulative Illness Rating Scale.

**Figure 2:**
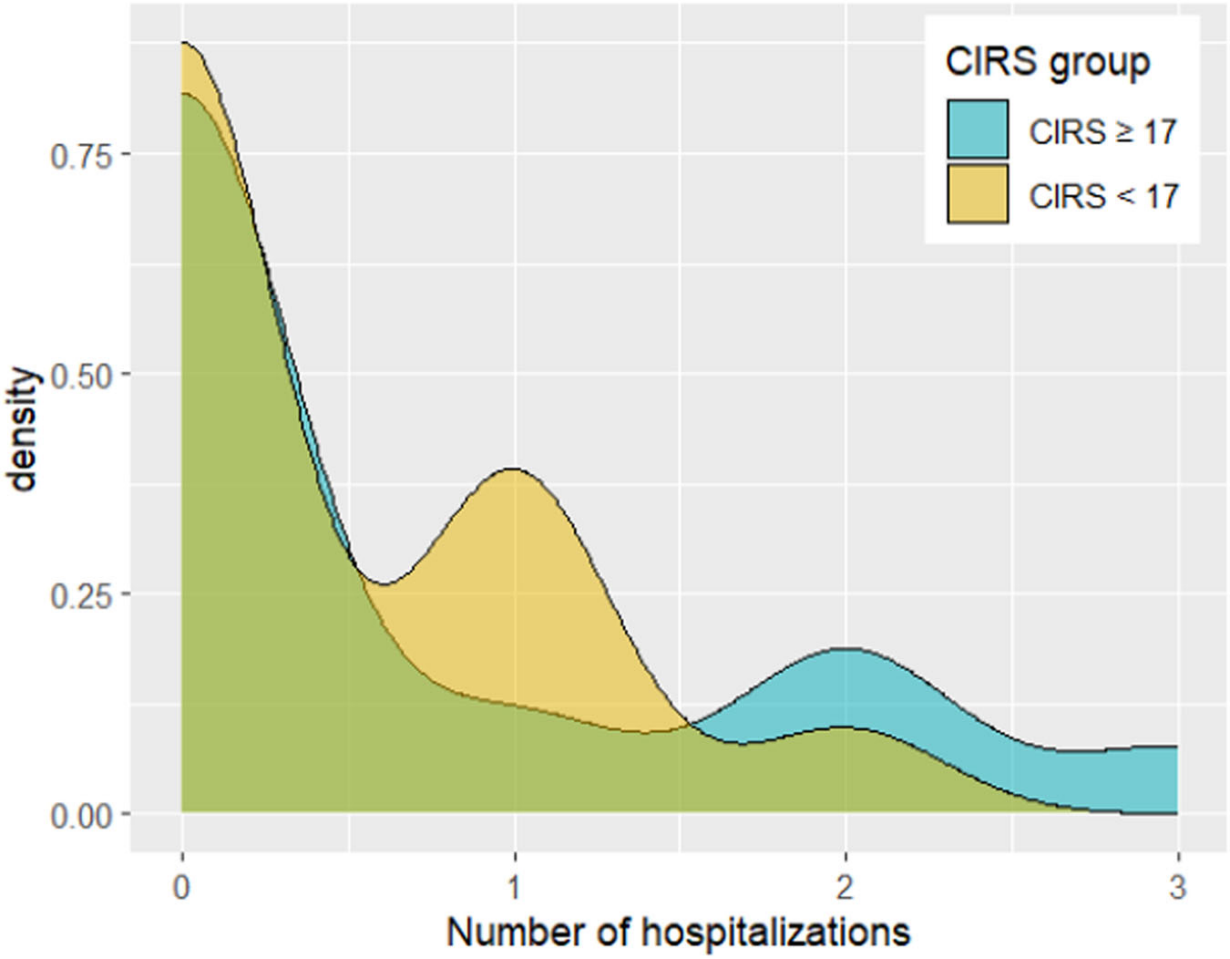
Density plot of the Number of hospitalizations for the two groups (CIRS ≥ 17 and CIRS < 17). CIRS, Cumulative Illness Rating Scale.

**Figure 3:**
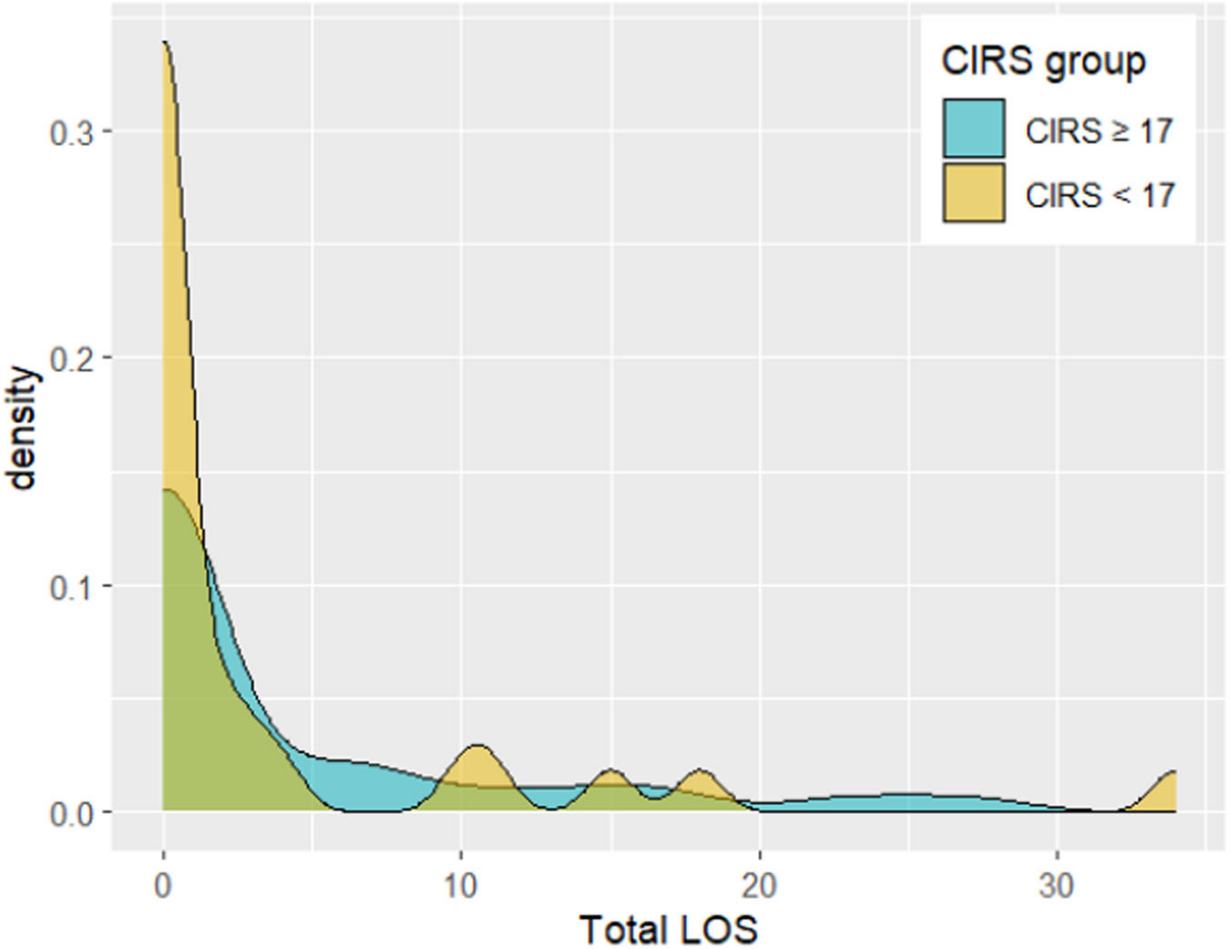
Density plot of the Total LOS for the two groups (CIRS ≥ 17 and CIRS < 17). CIRS, Cumulative Illness Rating Scale; LOS, length of stay.

**Figure 4:**
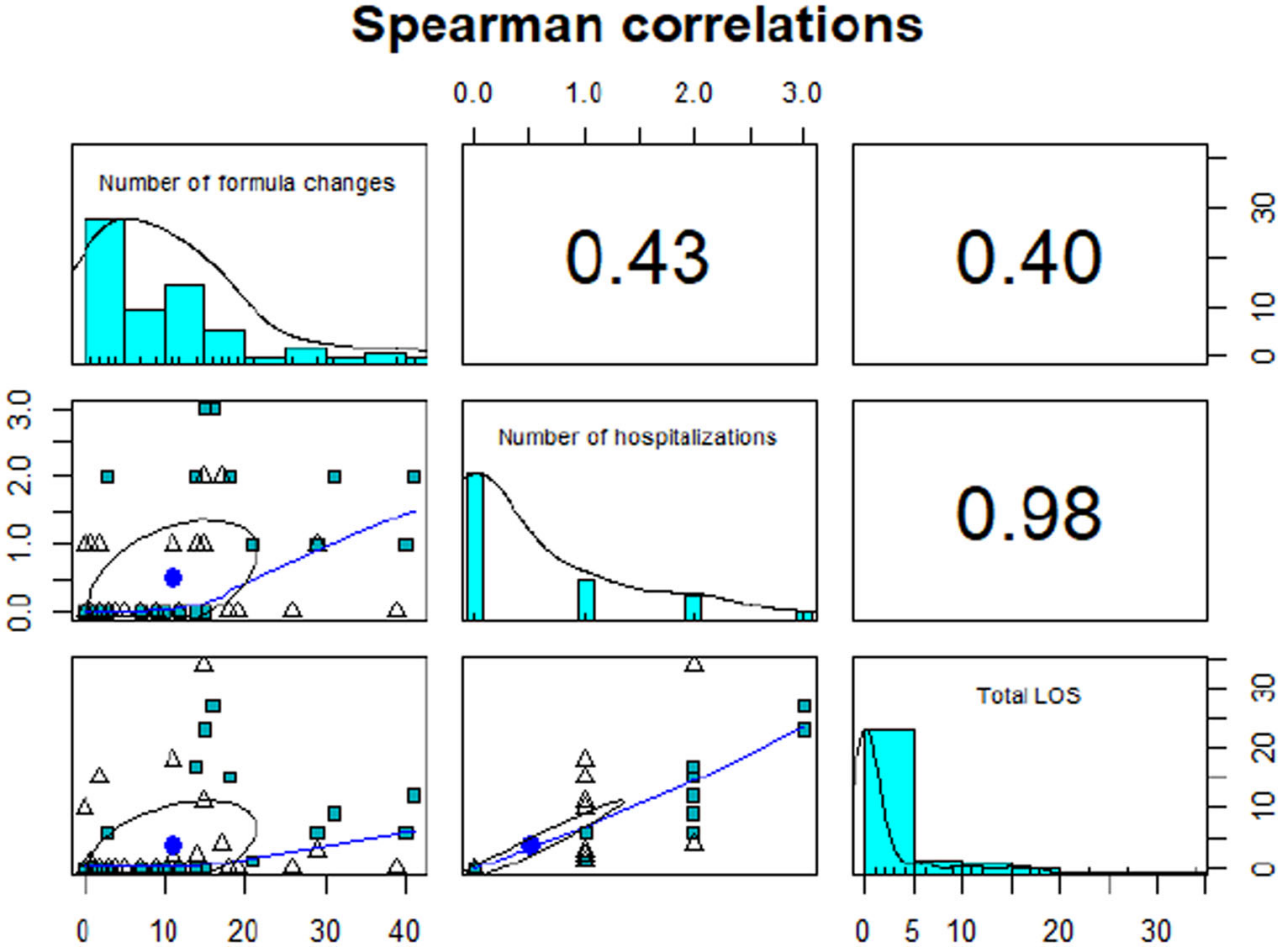
Correlation matrix plot showing Spearman correlations among the three variables of interest (formula changes, hospitalizations, and total LOS) for the entire sample of *N* = 60. LOS, length of stay.

Descriptive statistics were compiled for the variables of formula changes, hospitalization, and total LOS during the 90‐day study period in the two CIRS‐based groups (Table 2; Figures 1, 2, 3). A correlation matrix was obtained showing Spearman correlations among the three variables of interest (Figure 4).

**Table 2:** Descriptive statistics for variables between the two groups (CIRS ≥ 17 and CIRS < 17).

Variable	*M*	SD	Min	Median	IQR	Max	*n*
Formula changes							
CIRS ≥ 17	11.50	11.00	0	9.50	12.00	41	32
CIRS < 17	10.60	9.62	0	8.00	11.80	39	28
Hospitalizations							
CIRS ≥ 17	0.59	0.98	0	0	1.00	3	32
CIRS < 17	0.43	0.63	0	0	1.00	2	28
Total LOS							
CIRS ≥ 17	3.81	7.28	0	0	6	27	32
CIRS < 17	3.57	7.70	0	0	2.25	34	28

Abbreviations: CIRS, Cumulative Illness Rating Scale; IQR, interquartile range; LOS, length of stay; *M*, mean; Max, maximum; Min, minimum.

The CIRS score was normally distributed (Shapiro‐Wilk test: *W* = 0.98, *P* = 0.38). Wilcoxon rank sum tests (in Table 3) indicated that the two CIRS‐based groups (CIRS ≥ 17 and CIRS < 17) were not significantly different for each of the three variables: formula changes, hospitalization, and total LOS. The two‐sample *t* test results (Table 4) indicated that the groups (CIRS ≥ 17 and CIRS < 17) were significantly different based on mean CIRS scores. The negative binomial regression model (in Table 5) revealed that the number of hospitalizations was significantly different for the group CIRS ≥ 17 interacted with formula changes compared with the group CIRS < 17 interacted with formula changes. This implies that hospitalizations were significantly different at each level of formula changes for the group CIRS ≥ 17, whereas this is not the case for the group CIRS < 17.

**Table 3:** Wilcoxon rank sum test between the two groups: Cumulative Illness Rating Scale ≥ 17 and Cumulative Illness Rating Scale < 17.

Variable	Statistic (*W*)	*P* value	H0
Formula changes	449.5	0.9882	True location shift = 0
Hospitalizations	455	0.9081	True location shift = 0
Total LOS	439	0.8806	True location shift = 0

Abbreviation: LOS, length of stay.

**Table 4:** Two‐sample *t* test between the two groups: CIRS ≥ 17 and CIRS < 17.

Variable	Statistic (*t*)	df	*P* value	H0
CIRS score	10.91	58	<0.0001	True difference in means = 0

Abbreviation: CIRS, Cumulative Illness Rating Scale.

**Table 5:** Negative binomial regression model for regressing hospitalizations on CIRS group and formula changes.

	Estimate	SE	*z* value	Pr(>|*z*|)
CIRS ≥ 17: formula changes	0.06	0.02	3.48	<0.001
CIRS < 17: formula changes	0.03	0.02	1.27	0.204

*Note*: *P* < 0.05 is significant.

Abbreviation: CIRS, Cumulative Illness Rating Scale.

## DISCUSSION

In this pilot study, we showed that a multimorbidity tool could help identify HPN patients who required greater attention. Identifying such cases earlier could allow for proactive interventions, tailored resource allocation, and potentially improve outcomes and reduce HPN costs.

We found the CIRS to be well‐suited to HPN patient assessment. It was easy to learn and perform. It provided a single variable that could be used as a synopsis of the patient's multimorbidity and required only approximately 30 minutes of MNST time per patient at the end of each case presentation.

The CIRS data on our long‐term HPN patients showed a trend wherein a higher CIRS score was associated with greater resources for HPN and hospital care. This approach requires further study and validation.

There were several limitations to the current study. The available convenience study group was small, comprising only 60 patients, and the dataset contained some missing values. Furthermore, although the CIRS proved practical, its selection as the MMI tool for the HPN population may not be definitive. Other MMI tools, such as the CCI, could yield different results in this specific patient population.

An important practical consideration for the broader implementation of MMI scoring is the current lack of reimbursement by third‐party payers, which could impose additional costs on HPN organizations and thereby limit its use.

In summary, this pilot study provides preliminary evidence that an MMI tool can help identify HPN patients who require greater resource use and are at higher risk for adverse events. Although we acknowledge the limitations of our pilot and the need for further validation, our findings suggest that such an assessment could be a valuable addition to HPN patient management.

## AUTHOR CONTRIBUTIONS

Michael M. Rothkopf designed the study, served as principal investigator, and drafted and edited the manuscript. Mohan Pant performed the statistical analyses, created the figures, and edited the manuscript. Zachary Rothkopf reviewed the literature on MMIs, applied the Stirland guide to select the CIRS methodology, and edited the manuscript. Rebecca Brown participated in the MNST and calculation of CIRS scores on each study patient. Jamie Haselhorst participated in the MNST and calculation of CIRS scores on each study patient. Debbie L. Stevenson participated in the MNST and calculation of CIRS scores on each study patient. Andrew DePalma participated in the MNST and calculation of CIRS scores on each study patient. Michael Saracco participated in the MNST and calculation of CIRS scores on each study patient. All authors confirm that they had full access to all the data in the study and accept responsibility for the decision to submit for publication.

## CONFLICT OF INTEREST STATEMENT

None declared.

## Supporting information

CIRS calculation worksheet example 8‐6‐25.

## Data Availability

Data described in the manuscript will be made available upon request to the first author, pending application and approval.
