# Supplementary material for: Pilot study on the use of a multimorbidity index in patients receiving home parenteral nutrition
Source: Nutr Clin Pract. 2025 Sep 16;41(2):554–61. doi: 10.1002/ncp.70034 (PMC12982640; doi:10.1002/ncp.70034)
Supplement: Supplementary file 1 — CIRS calculation worksheet example 8‐6‐25. [file NCP-41-554-s001.pdf]

Cumulative Illness Rating Scale

Example Patient: 52 yo F with SBS, ↑creatinine, ↑LFTs, ↓Hb, murmur, depression

**Rating Score***	**0**	**1**	**2**	**3**	**4**
(description)	none	minor	moderate	severe	very severe
**System**					
cardiac	.	.	2	.	.
vascular	.	1	.	.	.
hematological	.	.	2	.	.
respiratory	0	.	.	.	.
otorhino-laryngological	0	.	.	.	.
ophthalmological	0	.	.	.	.
upper gastrointestinal	.	.	.	.	4
lower gastrointestinal	.	.	2	.	.
hepatic and pancreatic	.	.	.	3	.
renal	.	.	.	3	.
genitourinary	0	.	.	.	.
musculoskeletal, tegumenta	.	.	2	.	.
neurological	0	.	.	.	.
endocrine, metabolic, breas	0	.	.	.	.
psychiatric	.	.	2	.	.
Ratings Sub-totals	0	1	10	6	4
**Total CIRS score**	**21**				

* Rating of 0 indicates that there is no current problem affecting that system
* Rating of 1 indicates a mild current problem or a past significant problem
* Rating of 2 indicates a moderate problem requiring first-line therapy
* Rating of 3 indicates a severe problem that may be associated with significant disability or is hard to control
* Rating of 4 indicates an extremely severe problem, organ failure, or severe functional impairment.
